# Degradation of oil products in a soil from a Russian Barents hot-spot during electrodialytic remediation

**DOI:** 10.1186/s40064-016-1882-5

**Published:** 2016-02-24

**Authors:** Kristine B. Pedersen, Tore Lejon, Pernille E. Jensen, Lisbeth M. Ottosen

**Affiliations:** Department of Chemistry, University of Tromsø, The Arctic University of Norway, Postbox 6050, 9037 Langnes, Tromsø, Norway; Department of Civil Engineering, Arctic Technology Centre, Technical University of Denmark, Brovej Building 118, 2800 Lyngby, Denmark

**Keywords:** Electrokinetic remediation, PLS, PCA, Oil pollution, PAH

## Abstract

A highly oil-polluted soil from Krasnoe in North-West Russia was used to investigate the degradation of organic pollutants during electrodialytic remediation. Removal efficiencies were up to 70 % for total hydrocarbons (THC) and up to 65 % for polyaromatic hydrocarbons (PAH). Relatively more of the lighter PAH compounds and THC fractions were degraded. A principal component analysis (PCA) revealed a difference in the distribution of PAH compounds after the remediation. The observed clustering of experiments in the PCA scores plot was assessed to be related to the stirring rate. Multivariate analysis of the experimental settings and final concentrations in the 12 experiments revealed that the stirring rate of the soil suspension was by far the most important parameter for the remediation for both THC and PAH. Light was the second most important variable for PAH and seems to influence degradation. The experimental variables current density and remediation time did not significantly influence the degradation of the organic pollutants. Despite current density not influencing the remediation, there is potential for degrading organic pollutants during electrodialytic removal of heavy metals, as long as a stirred set-up is applied. Depending on remediation objectives, further optimisation may be needed in order to develop efficient remediation strategies.

## Background

Electrodialytic remediation (EDR) is a technology viable for removing heavy metals from polluted soil (Ottosen et al. 1997); the effect on organic pollutants has yet to be investigated. EDR is based on the principles of electrokinetic remediation (EKR) in which an electric field is applied directly to the soil and electrolysis reactions at the inert electrodes produces protons at the anode and hydroxyl ions at the cathode (Acar and Alshawabkeh 1993). An acidic front formed at the anode dominates the system, mainly due to higher effective ionic mobility of protons compared to hydroxyl ions (Acar and Alshawabkeh 1993). Transport processes in the soil under the influence of an electric field include electromigration (transport of ions in solution), electroosmosis (transport of fluid through pores), electrophoresis (transport of charged particles) and diffusion (Acar and Alshawabkeh 1993; Acar et al. 1993).

**Fig. 1:**
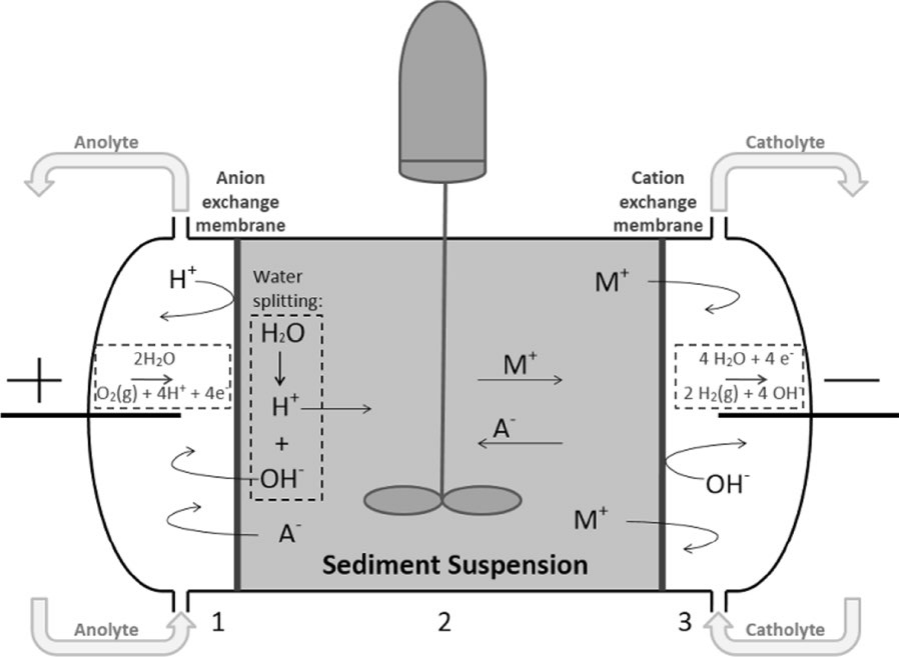
The 3-compartment EDR cell

The coupling of EKR with bioremediation has been shown to increase removal efficiencies of hydrophobic organic contaminants such as polyaromatic hydrocarbons (PAH) and total petroleum hydrocarbons (TPH) in soil/sediments (Dong et al. 2013; Gill et al. 2014; Kim et al. 2010; Li et al. 2012; Lohner et al. 2009; Niqui-Arroyo et al. 2006; Wang et al. 2013; Wick 2009). The bioavailability of hydrophobic contaminants is increased by advancing contact between the catabolically active microorganisms, nutrients, electron donors/acceptors and contaminants. Transport of nutrients and electron donors/acceptors is dominated by electromigration, while the transport of hydrophobic contaminants is dominated by electroosmosis (Gill et al. 2014; Lohner et al. 2009; Wick 2009). Microorganisms may be transported via electroosmosis and/or electrophoresis depending on their properties; e.g. weakly charged bacteria are primarily transported by electroosmosis (Wick 2009). Increasing the bioavailability of contaminants depends on the types of microorganisms as well as the physicochemical properties of the given chemical (Gill et al. 2014). Enhancement of the combined EK-biodegradation process includes the external introduction of nutrients, microorganisms that degrade the targeted contaminants and surfactants to increase the mobility of contaminants and/or microorganisms (Dong et al. 2013; Gill et al. 2014; Lohner et al. 2009). The electrolysis reactions (Fig. 1) may also be used to increase degradation adjacent to the electrodes, in which H_2_ acts as electron donors and O_2_ acts as electron acceptors (Lohner et al. 2009).

Many studies within EK-biodegradation have been based on spiked soils and reported high removal efficiencies of the spiked soils have not been possible to reproduce for historically polluted soil samples. However, removal efficiencies of up to 60 % of TPH in natural soils has been reported (Dong et al. 2013), while removal efficiencies of PAH are low. The addition of surfactants for the mobilisation of PAH has proven to increase removal efficiencies of aged contaminated soil/sediments to 30 % (Colacicco et al. 2010; Dong et al. 2013; Li et al. 2012; Niqui-Arroyo et al. 2006; Wang et al. 2013).

The influence of applying an electric field on microbial communities has mainly been assessed by regarding the contaminant degrading bacteria, and generally limited affect has been detected (Dong et al. 2013; Li et al. 2012; Lohner et al. 2009; Mena et al. 2014; Shi et al. 2008a, b; Wick et al. 2004), and may even stimulate microbial activity (Shi et al. 2008b). Negative impacts on microorganisms during EKR have however been observed and since these occurred adjacent to electrodes, this was attributed to changes in pH rather than the influence of the direct current (Kim et al. 2010; Schmidt et al. 2007). Wick et al. found that the electrodes may inhibit microbial communities if not physically shielded (Wick et al. 2004). In addition, chlorine and hydrogen peroxide can be generated in secondary electrode reactions and may inhibit microbial communities adjacent to the electrodes (Gill et al. 2014).

In EDR, ion exchange membranes are used to control transport between electrolytes and the polluted soil; in the 3-compartment cell as used in this study (Fig. 1), ions and compounds generated from reactions at the electrodes are prevented from entering the polluted soil. Acidification of the soil still occurs, mainly due to water splitting at the anion exchange membrane (Ottosen et al. 2000). Stirring the sediments has been shown to significantly improve removal efficiencies of heavy metals compared to the stationary cell set-up (Ottosen et al. 2012). Applying a stirred cell set-up may potentially influence the removal of organic contaminants due to increased aeration and mixing possibly resulting in increased bioavailability of the contaminants; Lima et al. for instance reported removal efficiencies of up to 79 % for PAH in a stirred set-up combined with use of surfactants (Lima et al. 2012).

Multivariate design and analysis provide tools for identifying trends and correlations in a system, including variable importance. A complete two-level factorial design contains all possible combinations of the settings, resulting in all variables as well as variable interactions being modelled. A fractional factorial design is constructed as a fraction of a complete factorial design, thus yielding less information as some variables will be confounded with other variables. It is however still possible to construct the design so that main effects and two-variable effects are only confounded with higher order interaction effects. Since higher order interaction effects are assumed to be negligible, the fractional designs can be used to obtain good estimates of the true main effects and the true two-variable interaction effects (Carlson and Carlson 2005). In most studies the identification and setting/tuning of the most important variables will be sufficient for obtaining satisfactory results but there are methods of extracting more information from the data, e.g. by employing the experimental variables as input in Projections onto Latent Structures (PLS) analysis.

In Projections onto Latent Structures (PLS), the quantitative relation between a descriptor matrix, X and a response matrix, Y is calculated. In this study PLS is hence used to establish the quantitative relation between experimental settings and final concentrations; the model subsequently used to retrieve the comparative influence of the experimental variables. PLS is based on projections providing the possibility of having more variables than objects. Other advantages of PLS include simultaneously modelling several responses, coping with collinearity between variables, noise in both the X and Y matrices, coping with moderate amounts of missing data (<20 %) and providing plots of the data compressed to fewer dimensions than the original dataset (Abdi 2010; Carlson and Carlson 2005; Trygg and Wold 2002; Wold et al. 2001).

In EDR studies, PLS has been used to identify the comparative importance of experimental variables (Pedersen et al. 2015c, d), determine sediment-specific optimal settings and has in addition been proven a good method for predicting experimental settings for new sediments when data from several sediments has been compiled (Pedersen et al. 2015b).

The objectives of this study were to investigate possible degradation of heavily oil polluted soil, including PAH compounds during EDR and in addition applying multivariate analysis to determine the influence of the experimental settings, including current density, remediation time, liquid–solid (L/S) ratio and light/no light.

## Experimental

### Description of site and experimental sample

The land plot Cape Knevatyi is a man-made part of Nikolsky Island located in the Northern Dvina River, Arkhangelsk, NW Russia. The land plot was constructed in 1966 with a size of approximately 60,000 m^2^ and has since been reduced to an area of 30,000 m^2^ due to erosion into the river. In the 1970s the land plot was used as storage site for oil-contaminated water, discharged into on-site pits from vessels. There are three ground pits with diameters of approximately 15–50 m located 20–100 m from the river. The oil pollution has been observed to have dispersed to the river bank which erodes into the river. The Northern Dvina River contributes 70 % of river water to the White Sea hence providing a direct path of pollution from Cape Knevatyi to the White Sea.

Pollution with TPH was observed to cover an area of approximately 2500 m^2^ and to depths of approximately 3 m. Hotspot areas were observed in the topsoil layer in the depths 0.5–1.5 m. Soil used in this study was sampled from hotspot at depths of 1.0–1.5 m by drilling (ø63 mm). The samples were kept cool during transport and were stored in a freezer until use/analysis.

### Analytical

*Major elements and heavy metal concentrations* (P, Al, Ca, Fe, K, Mg, Mn, Na, V, Cr, Cu, Ni, Pb, Zn) were measured based on digestion (Danish standard DS259). Sediment dried at 105 °C (1.0 g) and HNO_3_ (9 M, 20 mL) were autoclaved (200 kPa, 120 °C, 30 min). Solid particles were subsequently removed by vacuum filtration through a 0.45 µm filter and the liquid was diluted to 100 mL. Metal concentrations in the liquid were measured by Inductively Coupled Plasma-Optical Emission Spectrometry (ICP-OES).

*Organic components* (PAH, PCB, THC) were measured by ISO/DIS 16703 externally at a licensed laboratory, Eurofins in Moss, Norway.

*Carbonate* content was measured by treating dried sediment (5.0 g) with HCl (3 M; 20 mL) and the developed CO_2_ was measured volumetrically in a Scheibler apparatus, calibrated with CaCO_3_.

*Organic matter* was based on loss of ignition of dried sediment (2.5 g) being heated at 550 °C for an hour.

*pH (KCl)* Dried sediment (5.0 g) was agitated with KCl (1 M, 12.5 mL) for an hour and pH was subsequently measured using a radiometric analytical electrode.

*Conductivity* Dried sediment (5.0 g) was agitated with distilled water (25 mL) for an hour and the conductivity was subsequently measured using a radiometric analytical electrode.

*Chloride* content was measured by agitating sediment (10 g) dried at 40 °C with micropore water (40 mL) for 20 h. Solid particles were removed by 0.45 µm vacuum filtration and the chloride concentration was measured by ion chromatography.

*Total Carbon* (TC) and *Sulphur* (S) were measured by high temperature combustion. Dried sediment (0.5 g) was combusted (1350 °C) converting all carbon and sulphide into carbon dioxide and sulphur dioxide, respectively. The gasses were passed through scrubber tubes to remove interferences and the carbon dioxide and sulphur dioxide were measured by infrared detector.

*Nitrogen* (N) was measured by the Kjeldahl method. Dried sediment (1.0 g) was heated to 370 °C with H_2_SO_4_ (conc., 15 mL) and K_2_SO_4_ (7 g) until white fumes were observed (approx. 90 min) and subsequent to cooling 250 mL distilled water was added to the mixture. pH of the mixture was raised by adding NaOH (45 %) and subsequently the mixture was distilled and the vapours were trapped in HCl (15 %, 85 mL). The trapped vapour solution was subsequently titrated with NaOH (5 M).

*Grain size* was measured by wet sieving and dry sieving. Wet sediment (75 g), distilled water (350 mL) and Na_4_P_2_O_7_^.^10H_2_O (0.1 M, 10 mL) were agitated for 24 h. The slurry was then sieved through a 63 µm sieve and the fraction above 63 µm was subsequently dried and sieved for 15 min in a mechanical shaker using sieves with screen openings of 0.063, 0.080, 0.125, 0.25, 1.0 and 2.0 mm. The slurry fraction below 63 µm was transferred to Andreasen pipette for gravitational sedimentation. Stoke’s law was used for estimating the time required for particles to settle 20 cm and samples representing the sizes 40, 32, 16, 8, 4, 2 and 1 µm were measured.

### EDR experiments

#### Materials

The electrodialytic cell was manufactured from Plexiglass and consisted of 3 compartments; the centre compartment contained the polluted soil suspension and electrolyte liquids were circulated in the two adjoining compartments via external flasks (non-sealed). A schematic illustration of the cell design is shown in Fig. 1. The dimensions were; electrolyte compartment length 3.5 cm; centre compartment 10 cm; all compartments had inner diameter of 8 cm. Ion exchange membranes from Ionics (anion 204SZRAB02249C and cation CR67HUYN12116B) separated the electrolyte compartments from the centre compartment. NaNO_3_ (0.01 M) was used as electrolyte and was regularly adjusted to pH 2 by HNO_3_ (5 M). Electrolytes (350 mL) were circulated via Ismatec reglo pump with flow rate of 10 mL/min. Platinum coated titanium electrodes were used and a power supply (Hewlett Packard E3612A) maintained a constant DC current. A CATR14 motor with a stirrer consisting of plastic flaps (4 cm × 0.5 m) fastened to a glass rod ensured stirring of the soil suspension. No light conditions in selected experiments were obtained by covering the EDR cell in tin foil. The experiments were conducted at room temperature, around 20 °C.

After EDR experiments, the suspension liquid was decanted and organic and inorganic content in the sediment were analysed.

#### Design

The experimental design included the continuous variables current density, remediation time, stirring rate and liquid–solid (L/S) ratio and the discrete variable light/no light. Undertaking a complete two-level factorial design would entail conducting 2^5^ (32) experiments. By assuming that the interaction effects between 4 and 5 factors are negligible, the number of experiments can be reduced to a 2^5–2^ fractional design consisting of 8 experiments (no. 1–8). In addition, 4 experiments (no. 9–12) were conducted in the centre point of the experimental domain of the continuous variables. The experimental settings are presented in Table 1.

**Table 1:** Experimental design

Exp	Current density (mA/cm^2^)	Time (h)	Stirring rate (rpm)	L/S (mL/g)	Light
1	0	48	1300	4	Light
2	0.1	48	100	4	No light
3	0	432	100	4	Light
4	0.1	432	1300	4	No light
5	0	48	1300	2	No light
6	0.1	48	100	2	Light
7	0	432	100	2	No light
8	0.1	432	1300	2	Light
9	0.05	240	700	3	Light
10	0.05	240	700	3	No light
11	0.05	240	700	3	Light
12	0.05	240	700	3	No light

### Multivariate modelling

In this study, SimcaP11 was used for PCA and PLS modelling based on the experiments in Table 1. In calculation of a PCA model, the X matrix consisted of the concentrations of PAH compounds before and after the experiments. In the PLS models, the X-matrix consisted of the experimental settings and the Y matrix consisted of the final concentrations of organic pollutants. In order to include the discrete variable light/no light in the models, the two conditions were arbitrarily assigned a value of 1 or −1.

Correlation factors (R2X and R2Y) and predictive powers were used to evaluate the reliability of the multivariate models. R2X is a measure of the fraction of the X matrix explained by the model while R2Y is a measure of the fraction of the Y matrix explained by the model and Q2 is an estimate of the reliability of the model calculated by cross-validation. Variable Importance in the Projection (VIP) plots are absolute values that present the importance of each parameter in a given model with respect to its correlation to all the responses (Y) and to the projection (X) and were used to assess variable importance in the remediation. Parameters with VIP values above 1 are considered significant for explaining the responses; VIP values between 0.5 and 1 indicate a moderate influence on the model and parameters with VIP values below 0.5 have a low influence on the model. Contour plots were used to assess whether important variables had positive/negative influence on the models.

## Results and discussion

### Soil characteristics

The soil used for the EDR experiments exhibited low content of carbonate and organic matter indicating a low buffer capacity (Table 2). The concentrations of metals were also low, which may be due to the low content of clay and silt (<2 %) in the soil.

**Table 2:** Sediment characteristics

Characteristic	Units	Value	Russian QC
Carbonate	%	0.3 ± 13 %	
Organic matter	%	2.8 ± 8 %	
Total carbon	%	2.5 ± 2 %	
Total sulphur	%	0.06 ± 5 %	
Nitrogen	%	0.03 ± 2 %	
pH		3.9 ± 1.5 %	
Conductivity	mS/cm	0.08 ± 6 %	
Chloride	mg/kg	8.6 ± 30 %	
*Grain size*			
Clay	%	0.6	
Silt	%	1.1	
Sand	%	97.9	
Rubble	%	0.4	
Al	mg/kg	830 ± 9 %	
Ba		33 ± 39 %	
Ca		190 ± 22 %	
Fe		1650 ± 7 %	
K		150 ± 20 %	
Mg		140 ± 20 %	
Mn		16 ± 6 %	
Na		42 ± 12 %	
V		4.9 ± 27 %	
As		2.0 ± 34 %	2
Cd		0.1 ± 34 %	0.5
Cr		3.2 ± 26 %	6
Cu		5.7 ± 16 %	3
Ni		3.6 ± 19 %	4
Pb		6.0 ± 24 %	32
Zn		20 ± 17 %	23
Acenaphtene		0.74 ± 40 %	
Acenaphtylene		0.02 ± 40 %	
Anthracene		0.33 ± 40 %	
Benzo(a)anthracene		0.28 ± 40 %	
Benzo(a)pyrene		0.12 ± 40 %	0.02
Benzo(b)fluranthene		0.21 ± 40 %	
Benzo(ghi)perylene		0.05 ± 40 %	
Benzo(k)fluoranthene		0.06 ± 40 %	
Dibenzo(a,h)anthracene		<0.020	
Phenanthrene		2.6 ± 40 %	
Fluoranthene		0.28 ± 40 %	
Fluorene		1.1 ± 40 %	
Indeno(1,2,3-cd)pyrene		0.02 ± 40 %	
Chrysene		0.95 ± 40 %	
Naphtalene		1.3 ± 40 %	
Pyrene		2.1 ± 40 %	
PAH16		10	
PCB		n.d.	
C_5_-C_8_		<5.0	
C_8_-C_10_		130 ± 30 %	
C_10_-C_12_		890 ± 30 %	
C_12_-C_16_		77,00 ± 30 %	
C_16_-C_35_		13,000 ± 30 %	
THC, total		22,000	1000

Russian soil quality criteria (QC) for inorganic and organic priority substances are included

The concentrations of hydrocarbons were high, especially the heavier fractions C_12_–C_35_, and the total concentration of hydrocarbons were equivalent to more than 20 times the Russian soil quality criteria. During the sampling, a strong odour of oil products and free phase oil in the subsurface water in the drilling hole was observed. The sampled soil did not contain large amounts of peat or other organic material, which excludes contamination from naturally occurring hydrocarbons, indicating that the high hydrocarbon content is related to oil pollution. The soil had elevated concentrations of PAH exemplified by benzo(a)pyrene which had a sixfold concentration compared to soil quality criteria. No PCB (7 congeners) was detected and the concentrations of heavy metals are low and may be naturally occurring rather than due to anthropogenic sources. The targeted pollutants in this study were hydrocarbons and PAH components. The PAH component, dibenzo(a,h)anthracene, and the THC fraction C_5_–C_8_, were not detected and are not included in further analysis of the experiments.

Ratios of selected PAH components have been widely used to identify specific sources and although results may be ambiguous, they provide indicative values (Countway et al. 2003; Feng et al. 2014; Pedersen et al. 2015a; Soclo et al. 2000; Yunker et al. 2002). In cases of PAH pollution from several sources, the ratios may be used for evaluating predominant sources. The ratio of *anthracene/(anthracene and phenanthrene)* has previously been used for determining petrogenic (<0.1) and pyrogenic (>0.1) sources (Soclo et al. 2000). In this study the ratio was 0.1 and hence in the borderline of petrogenic and pyrogenic sources. The PAH ratios *fluoranthene/(fluoranthene and pyrene)* and *indeno(123*-*cd)pyrene/(indeno(1,2,3*-*cd)pyrene and benzo(ghi)perylene)* have been used to indicate whether pyrogenic sources originate from liquid fossil fuel combustion (0.4–0.5/0.2–0.5) or coal/biomass combustion (>0.5/>0.5) (Feng et al. 2014). In this study these ratios were 0.1 and 0.3, respectively, indicating that the source to be combustion of liquid fossil fuel. It is hence highly likely that the PAH pollution found at the site is related to the oil pollution at the site.

### Evaluation of EDR experiments

During the EDR experiments, high voltages were registered, occasionally above 100 V, in experiments no. 2, 4, 6 and 8, and employing a current density of 0.1 mA/cm^2^ was the limit of the equipment used. Low and steady voltages, generally below 2 V, were observed in the centre point experiments (no. 9–12). The reason for the fluctuations and high levels of voltage in the experiments conducted at 0.1 mA/cm^2^ may be due to the high concentration of oil products in the soil suspensions (Table 2) resulting in a low conductivity. During all experiments, thick dark brown/black oil was formed in the sediment compartment and this was hypothesized to be due to biodegradation of the original oil in the soil.

The pH in the experiments fluctuated between 2 and 4, with no clear indication of the influence of current. There was in addition no clear pattern in the increase and/or decrease in the pH during the experiments. Since concentrations of Al, which can act as a buffer around pH 4, was low, this may indicate that the high content of oil acted as buffer in the soil.

The concentration of some of the elements appeared to decrease during the experiments and in order to ascertain whether the observed differences were statistically significant, one factor variance analysis was undertaken. The final concentrations of Al, Ca, Fe, Mg, Ni and Zn were significantly lower after remediation experiments, while the F values of Ba, K, Mn, Na, As, Cd, Cr, Cu and Pb were below the critical value (3.98). It was not possible to determine whether the metals removed stemmed from the oil or were naturally occurring in the soil matrix.

The removal efficiencies of total THC in the 12 experiments were 30–70 % (Fig. 1). The highest removal efficiency was observed in experiment 8 (432 h; 0.01 mA; 2 mL/g; 1300 rpm; light) and the lowest removal efficiency was detected in experiment 3 (432 h; no current; 4 mL/g; 100 rpm; light). Apart from experiment 11, the removal efficiencies appear to be higher in the experiments conducted at the centre point of the continuous variables. Microbial communities could be inhibited by high voltage gradients (Gill et al. 2014; Lohner et al. 2009; Wick 2009), observed in experiments 1, 3, 5, and 7; the higher removal efficiencies in the centre experiments with low voltage gradients, may be an indication of such an effect. The difference between the centre point experiments and the experiments in which no current was applied could be an effect of microbial degradation induced by the electric field, in line with previous findings (Dong et al. 2013; Kim et al. 2010).

Removal of the heavier THC fractions (C_12_–C_16_ and C_16_–C_35_) and the total THC followed the same trend (Fig. 1a) and is likely due to more than 95 % total THC being found in these fractions. Even though large fluctuations were observed in the lighter fractions, this did not affect the total THC removal as the initial concentrations were low. In general, higher removal efficiencies of the lighter fractions (C_8_–C_10_ and C_10_–C_12_) were observed, in line with results of an EK study of diesel polluted soil, in which the highest degradation of the C_10_–C_16_ fraction was reported (Kim et al. 2010). In the present study, degradation of the lighter fractions appears to be related to stirring rate; the highest removal efficiencies observed at medium–high stirring rates (experiments 1, 4, 5, 8, 9, 11 and 12). In experiments 6, 7 and 10 removal efficiencies of the lighter fractions were lower compared to the total removal. In experiments 6 and 7 the low stirring rates and low L/S ratios were applied indicating that not enough aeration was provided to remove the lighter fractions. The difference in removal efficiencies between experiment 10 and the other 3 experiments conducted in the centre point of the continuous variables may indicate an inhomogeneous distribution of the oil pollution in the soil.

**Fig. 2:**
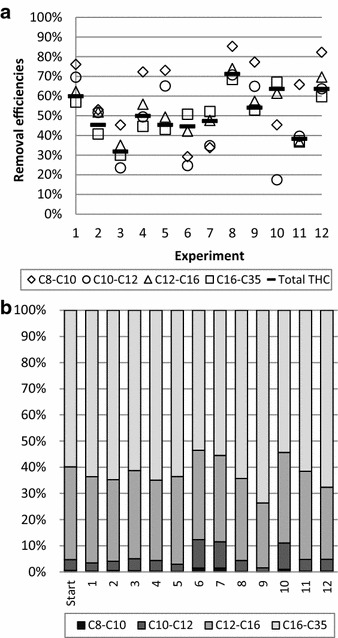
**a** Removal efficiencies of the THC fractions as well as the sum of THC removal and **b** distribution of the THC fractions before (start) and after each of the 12 experiments

Despite the variation in the removal efficiencies depending on the experimental settings, the distribution of the THC fractions were similar after the experiments; the exceptions being experiment 6, 7, 9 and 10 (Fig. 2b). In experiment 9, the deviation is related to relatively larger removal of lighter fractions; albeit assessed to be within the experimental deviation. The distribution patterns of experiments 6, 7, and 10 are in line with observations of the removal efficiency trends of the different fractions revealing relatively less removal of the lighter fractions compared to the other experiments. Based on the observations of removal efficiencies and differences in trends and distribution of the THC fractions in the experiments, it was at this point not possible to deduce which parameters had the highest influence on the remediation (vide infra).

**Fig. 3:**
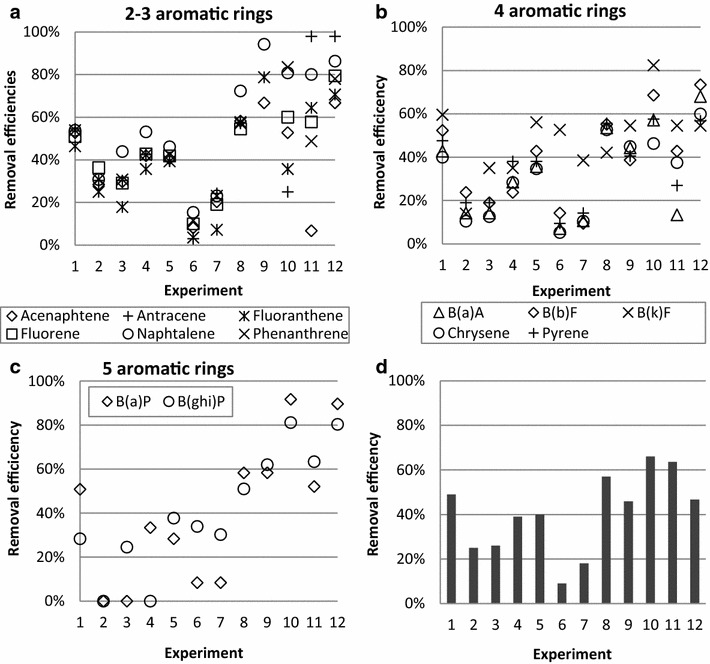
Removal efficiencies of PAH components; **a** containing 2–3 aromatic rings, **b** containing 4 aromatic rings, **c** containing 5 aromatic rings and **d** Sum of PAH16. *B(a)A* benzo(a)anthracene, *B(b)F* benzo(b)fluoranthene, *B(k)F* benzo(k)fluoranthene, *B(a)P* benzo(a)pyrene; and *B(ghi)P* benzo(ghi)perylene

The removal efficiencies of total PAH16 were 10–65 % (Fig. 3d) with the highest removal efficiencies (>40 %) observed in experiments 1, 8 and the centre point experiments. Differences in the removal trends of the different PAH components were however observed and could be related to the number of aromatic rings of the PAH (Fig. 3a–c). For the PAH containing 2–3 aromatic rings, the highest removal efficiencies were generally observed in experiments with medium–high stirring rates (1, 4, 5, 8–12). The removal efficiencies of these components were generally similar; fluoranthene, with the highest molecular weight in the group, consistently had the lowest removal efficiencies. Larger variations in the removal efficiencies were observed for PAH compounds containing 3–4 aromatic rings; although generally following the same trend between the experiments. Benzo(k)fluoranthene deviated the most from the general trend and was effectively removed. The deviations may be related to the low initial concentrations resulting in relatively larger errors in analyses. Apart from benzo(k)fluoranthene, the highest removal efficiencies of the PAH compounds with 3–4 aromatic rings were observed in experiments 1 and 8–12. For the PAH compounds containing 5 aromatic rings there was no clear trend in removal efficiencies. The highest removal efficiencies of benzo(a)pyrene was observed in the experiments with medium–high stirring rates (1, 4, 5, 8–12). The generally higher variation in removal in the centre point experiments may indicate inhomogeneous distribution of PAH in the soil.

**Fig. 4:**
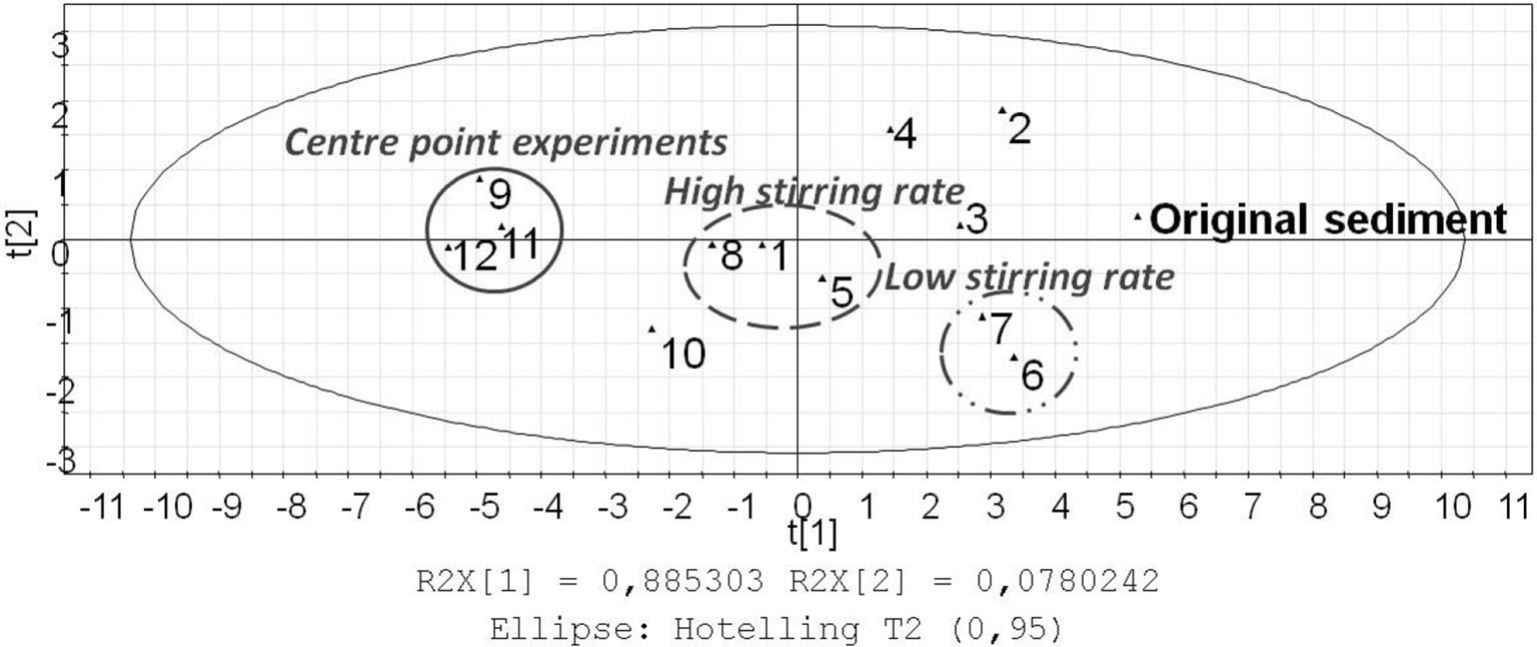
PCA scores plot of PAH component concentrations before (original sediment) and after experiments 1–12

In order to assess the distribution of PAH components before and after the experiments, a PCA model was calculated. The model explained 96 % (R2X) of the variation (Q2 92 %). The scores plot revealed different distribution of the PAH components after the experiments and were to some degree clustered according to the experimental settings (Fig. 4). The centre point experiments, apart from experiment 10, which was previously shown to deviate, were clustered in the left side of the scores plot. The experiments run at high stirring rates (apart from experiment 4) were clustered in the centre part of the scores plot. Experiments 6 and 7 had the lowest removal efficiencies and were also located close to one another. The PCA plot hence indicates that stirring rate may influence the remediation; multivariate investigations are however necessary to confirm these observations.

### Variable importance

In order to determine variable importance in the remediation, two PLS models of the final concentrations in the experiments were calculated:

Model 1: PAH components were inserted as responses.

Model 2: The fractions of hydrocarbons were inserted as responses.

Model 1 had a correlation factor, R2Y, of 0.89 and a predictive power, Q2, of 0.69 and was hence assessed as a good and stable model suitable for evaluation of variable importance in the experiments. Model 2 had a R2Y value of 0.62 and Q2 value of 0.26 and was hence a more instable model, but was none the less assessed as having an indicative value of variable importance.

**Fig. 5:**
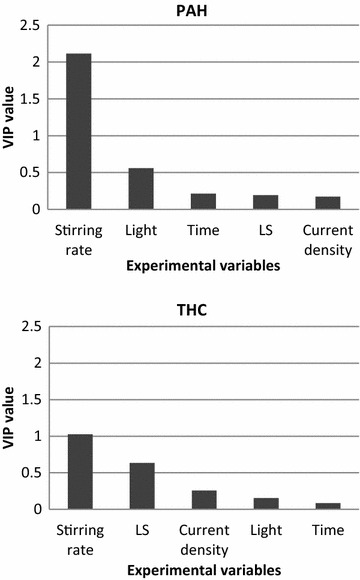
Summary of VIP plots for PAH and THC. High VIP values have the highest influence on the remediation

For both the degradation of PAH components and hydrocarbon fractions, as indicated by the preliminary assessments, the stirring rate was with VIP values above 1 by far the most important parameter (Fig. 5). Light appears to have a relatively higher influence on the degradation of PAH components than the hydrocarbon chains; the effect (VIP value ~0.6) however not as significant as the stirring rate. The L/S ratio had a relatively higher influence (VIP value ~0.6) on the degradation of hydrocarbons; coefficient plots of the four hydrocarbon fractions revealed that a low L/S ratio increased removal efficiencies. This may be an indication of a limiting amount of suspension liquid for increasing bioavailability of the hydrocarbons.

For both PAH components and the THC fractions, current density and time had low influences on the final concentrations (VIP values below 0.5), indicating that the EDR process itself did not significantly affect the degradation of organic contaminants within the experimental domain studied. Previous studies found that applying current to soil may stimulate microbial activity and enhance cell respiration, mainly attributed to production of oxygen at the anode (Kim et al. 2010); however this effect was not detectable in this study, which could be due to the anion exchange membrane preventing introduction of oxygen produced at the anode, or that aeration of the soil suspension by stirring rate introduced more oxygen than was produced at the anode. In an EKR coupled with bioremediation study, the initial fast degradation rate of PAH, was followed by a slower degradation rate (Li et al. 2012). In this study, the fast degradation may have occurred within the first 48 h, after which the influence of time on the removal efficiencies is negligible. In addition, the electrodialytic process may have depleted the soil suspension of nutrients. The pH in the soil suspensions did not decrease during EDR; microbial communities adapted to the initially high concentrations of oil were hence not inhibited by an increasing acidification during treatment and may be a reason for the high removal efficiencies achieved. From this study it is however not possible to assess the relative relation between bioremediation, volatilisation of oil compounds and transfer of pollutants to the water phase. Nonetheless, since the stirring rate has a significantly higher influence than light, this indicates that bioremediation and/or volatilisation was more significant than degradation by photolysis.

## Conclusions

The study indicated that simultaneous removal of organic pollutants during electrodialytic removal of heavy metals is possible. Depending on the remediation objective; e.g. if the organic pollutant concentrations should meet soil quality criteria, remediation strategies may include further optimisation steps to obtain these. For future design of simultaneous electrodialytic removal of heavy metals and organic pollutants it appears imperative to apply stirred set-ups and further optimisation could be achieved by stimulating further bioremediation, e.g. by adding nutrients and/or microorganisms.
